# Supplementary material for: Efficient and self-adaptive in-situ learning in multilayer memristor neural networks
Source: Nat Commun. 2018 Jun 19;9:2385. doi: 10.1038/s41467-018-04484-2 (PMC6008303; doi:10.1038/s41467-018-04484-2)
Supplement: Supplementary file 1 — Supplementary Information [file 41467_2018_4484_MOESM1_ESM.pdf]

Supplementary Information

Efficient and self-adaptive in-situ learning in multilayer memristor neural networks

Li et al.

Supplementary Figures 1-14;

Supplementary Table 1;

Supplementary Note 1.

Supplementary Figures

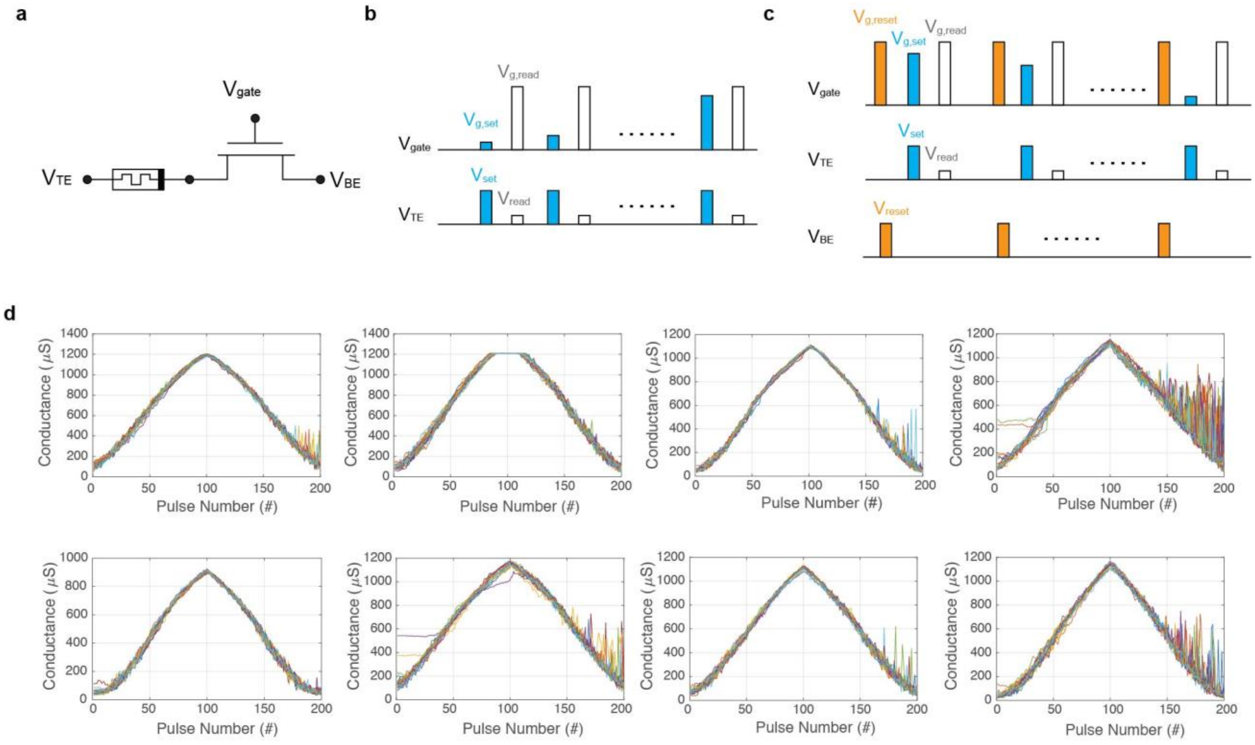

Supplementary Figure 1. Linear memristor conductance response to applied pulses. a, One transistor and one memristor connected in series to form the 1T1R structure. The bottom electrode of the memristor is connected to the drain of the transistor. **b,** The pulses applied to obtain conductance increases (about 500 µs in width) and **c,** for conductance decreases (about 5 µs). **d,** Evolution of conductance for eight random selected responsive devices

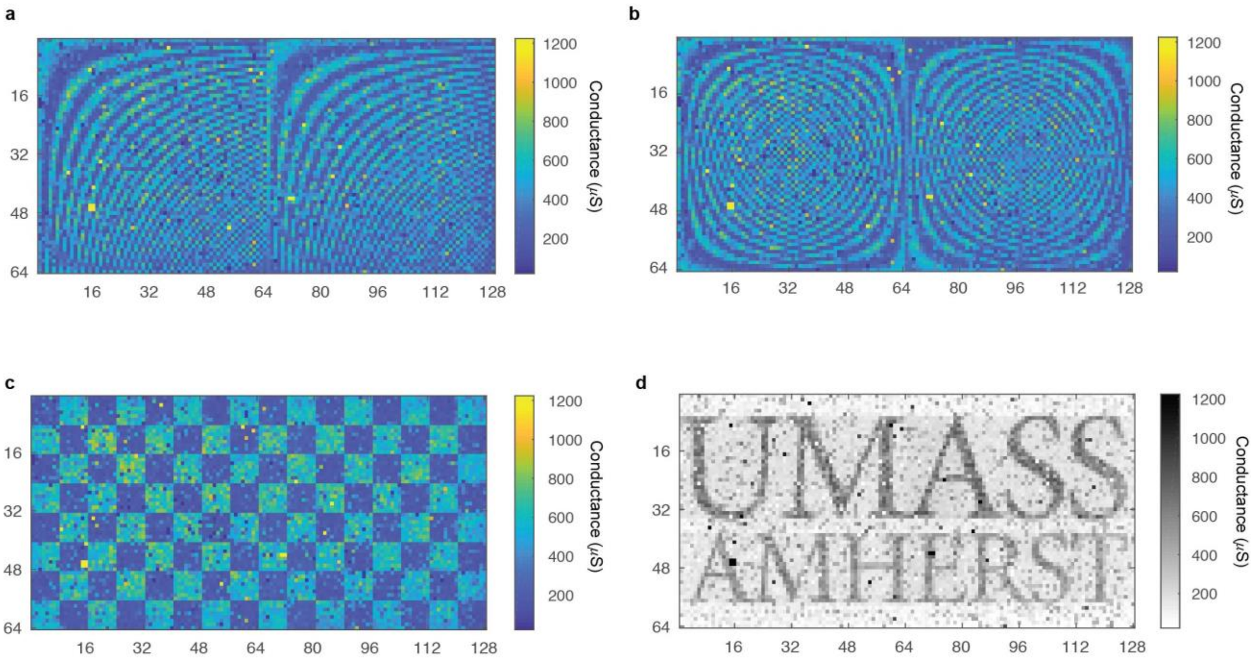

Supplementary Figure 2. Memristor array programming with a single pulse on each device. a, discrete cosine transformation (DCT) matrix pattern. **b**, discrete Fourier transformation (DFT) matrix pattern. **c**, checkerboard. **d**, UMASS AMEHRST wordmark in greyscale.

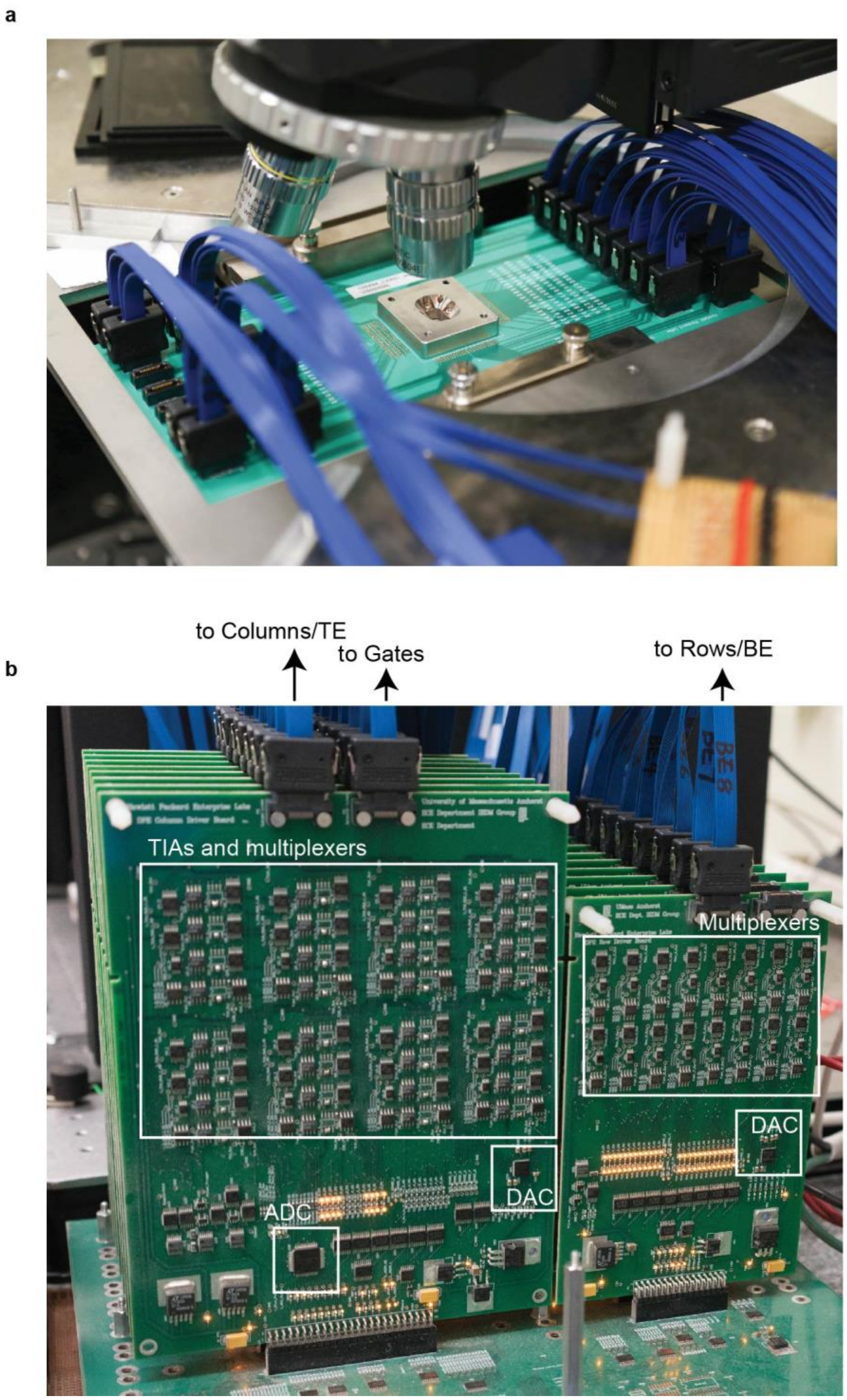

Supplementary Figure 3. Photograph of the printed circuit boards (PCBs). **a**, The probe card contacting the 1T1R chip under measurement. The high-speed cables (seen in blue) are connected to the external PCB based measurement systems. **b**, Column boards are shown on the left while row boards on the right. These PCBs communicate with a computer through a microcontroller, taking digital input vectors and supplying corresponding pulses of analog voltage signals to all rows simultaneously while sensing and rapidly sampling the currents at each column.

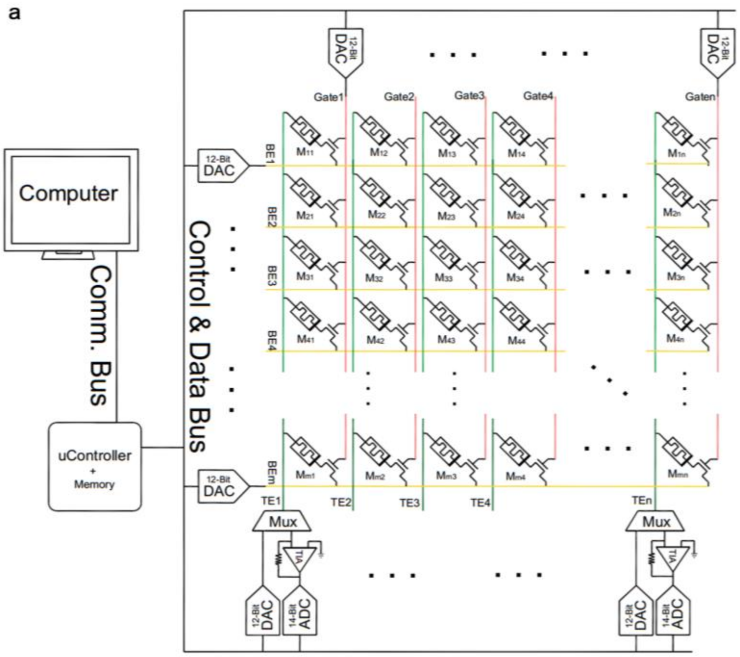

Supplementary Figure 4. Circuit diagram of the measurement system. MATLAB script with graphic user interface (GUI) running on a general-purpose computer is used to communicate with the microcontroller through a serial port for various applications. The microcontroller controls all the circuit components on the measurement system, such as DACs (Digital-Analog Converters) for generating driving pulses and ADCs (Analog-Digital Converters) for collecting the measurement data. Note that voltages applied on either rows or columns on the memristors crossbar, or gates on the transistors are generated in parallel by different DACs that located on the rows and columns boards. Four transimpedance amplifiers (TIAs) with different gains are attached to each column to cover 4 orders of magnitude current ranges, but only one TIA is chosen during the operation depending on the output current level. During a read or vector-matrix multiplication (VMM) operation, small voltages (representing input vectors) are applied on the corresponding rows (marked as BEs) by the DACs. Currents (representing output vectors) are read out by the TIAs and ADCs on columns (marked as TEs). The readout currents are processed in the microcontroller, and/or sent back to the general-purpose computer. The write operation is similar to the reading. Positive voltages can be applied on selected rows for reset or selected columns for set, while all other unselected terminals can be grounded, floated or biased with an arbitrary voltage value.

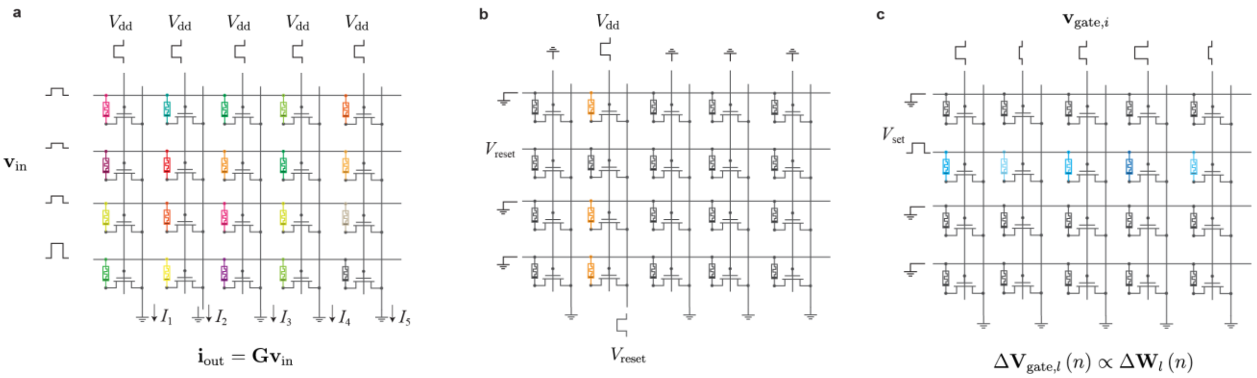

Supplementary Figure 5. Illustration of proposed 1T1R operation by electrical pulses. a, The matrix multiplication is performed by applying the input voltages (the amplitude of them represents the vector values) on the row wires, and reading the current on the column wires representing the output current. All the transistors are turned on by applying synchronized pulses on the word-line wires, making the 1T1R array a pseudo-crossbar. **b,** Parallel-reset scheme. A reset pulse is applied to the bottom electrodes of an entire column simultaneously, while the top-electrode voltages are used to control which devices are reset. **c,** Parallel-set scheme. Pulses are applied to the top electrodes of an entire row simultaneously, while the gate voltages vary along the row.

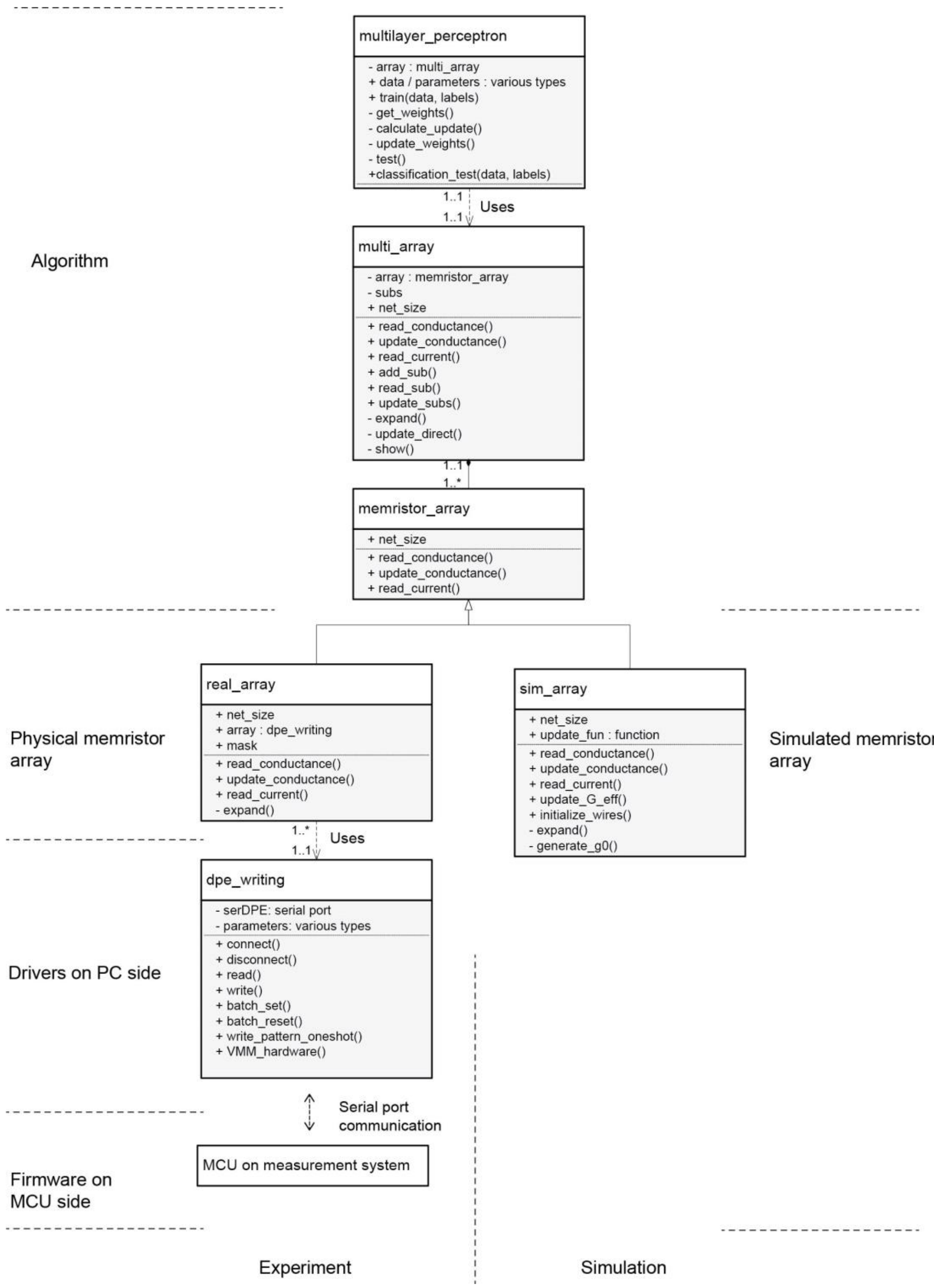

Supplementary Figure 6. UML class diagram of the algorithm. The experiment and the simulation share the exactly same upper level algorithm. In the simulation, we use the measurement data from the physical array, such as memristor conductance limit, memristor conductance-transistor gate voltage relation, etc., and consider some random noises and unresponsive devices for analyses. The firmware of the MCU is written in C language, and all others are in MATLAB.

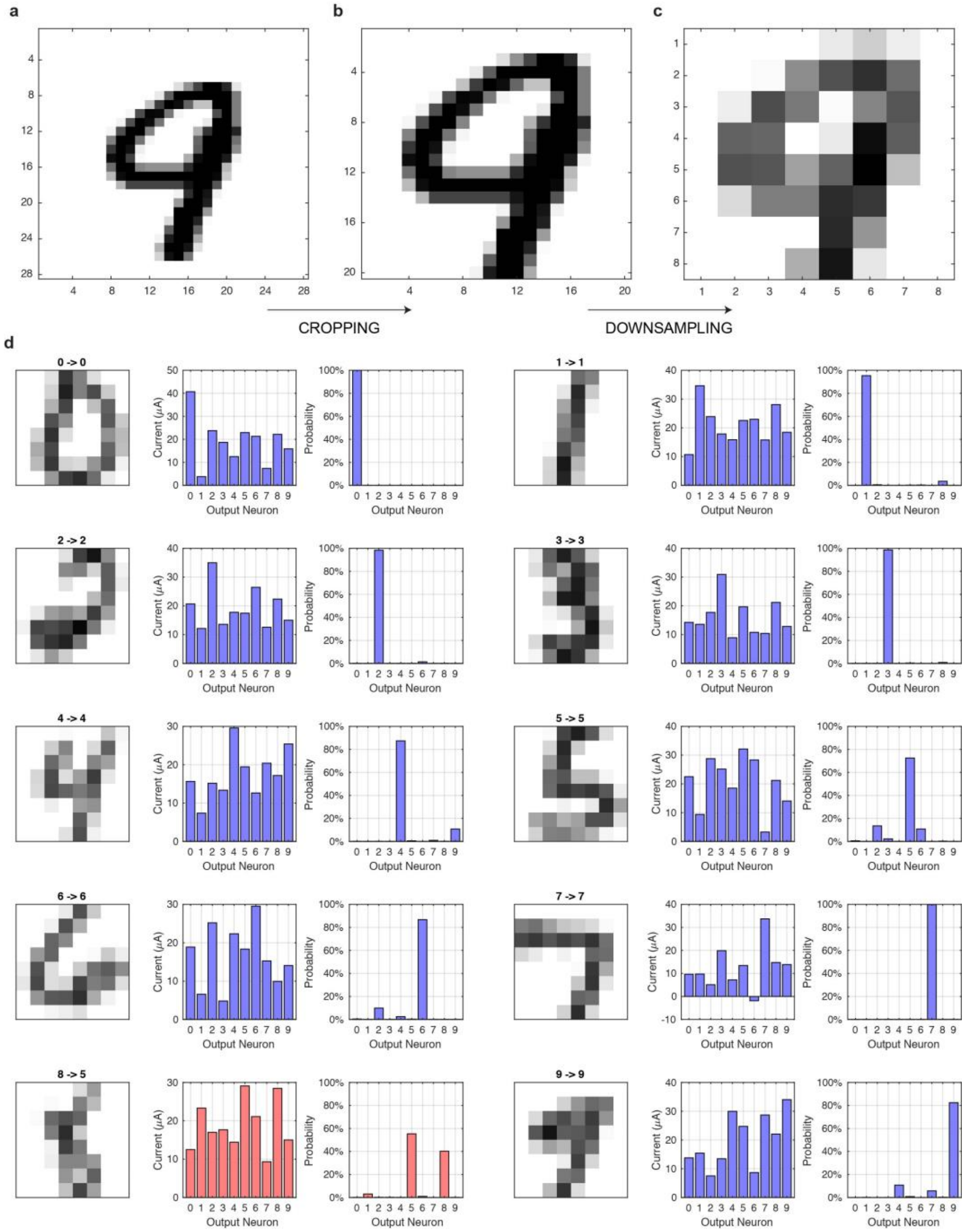

Supplementary Figure 7. Extended data for MNIST inference tests. **a-c**, The pre-processing of original MNIST images to fit our array dimension. The original images (28×28) (A) were cropped to keep the central 20×20 region, and then down-sampled to 8×8 with bicubic interpolation. The cropped and down-sampled images were converted to unrolled voltage vectors as input. **d**, Additional ten typical image inference results, with nine correctly classified (blue colored) and one misclassified (red colored, correct label is 'eight', while the crossbar outputs indicate 'five'). The raw output currents and Bayesian probabilities produced by the network for each image are also shown. More data is shown in *Supplementary Movie 2* and *Supplementary Movie 3*.

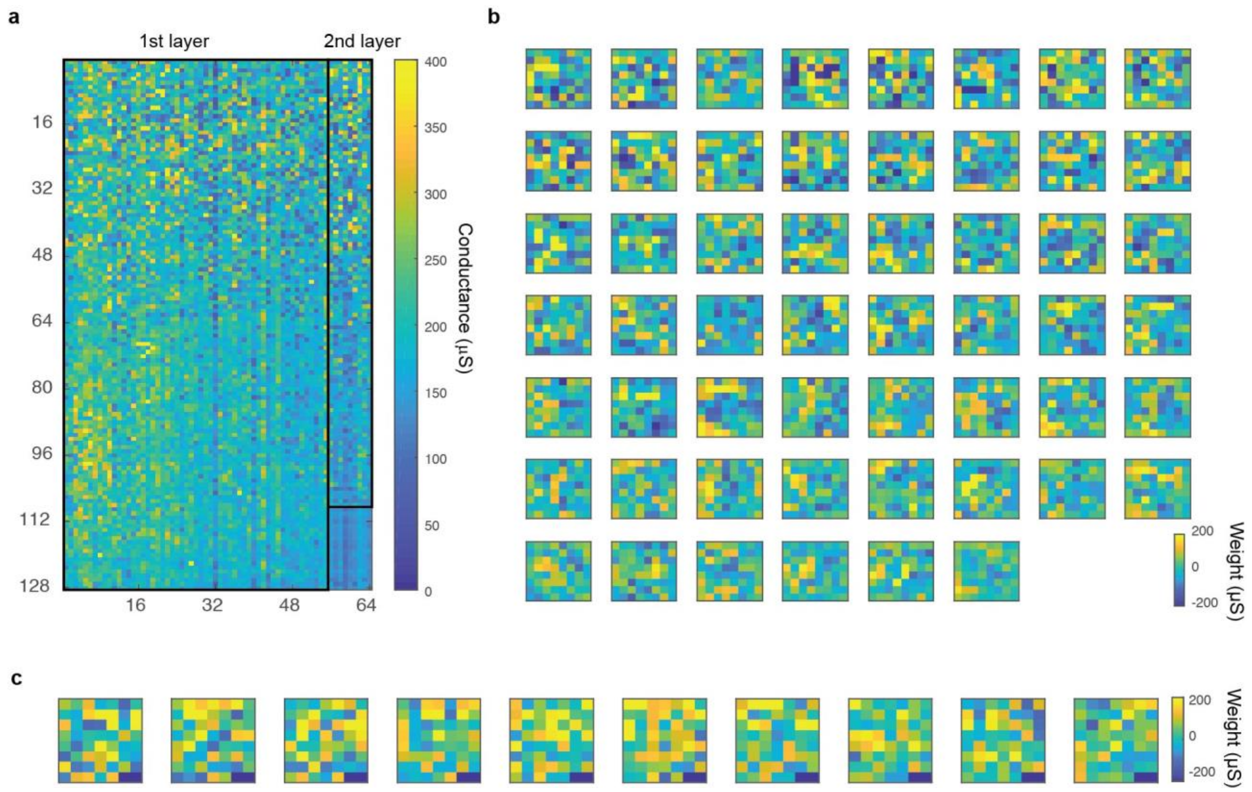

Supplementary Figure 8. Conductance and weights after training. **a**, The read out conductance map after training on 60,000 MNIST images. The conductance is read with an identity matrix (voltage inputs) as described in *Method: S5*. **b**, The first-layer weights after training on 80,000 MNIST image samples. Each square represents all synapses connected to one hidden neuron, with each pixel representing a single synapse. Each weight is the difference of the conductances between two memristors. The pixels are arranged to match the pixels of the input images. **c**, the second-layer weights after training. These synapses connect the hidden neurons to the output neurons. The pixels in each square are arranged to match hidden neurons in (b), while the lower right two are empty. The evolution of these weights during training is shown in *Supplementary Movie 1*.

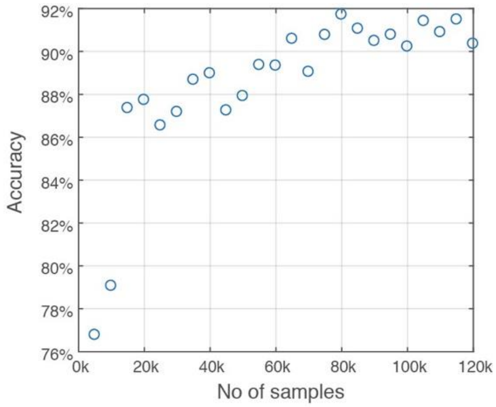

Supplementary Figure 9. Classification test on the entire separate testing set after training on every 5,000 samples. The testing accuracy increases as the number of training samples increases, saturating at around 91%.

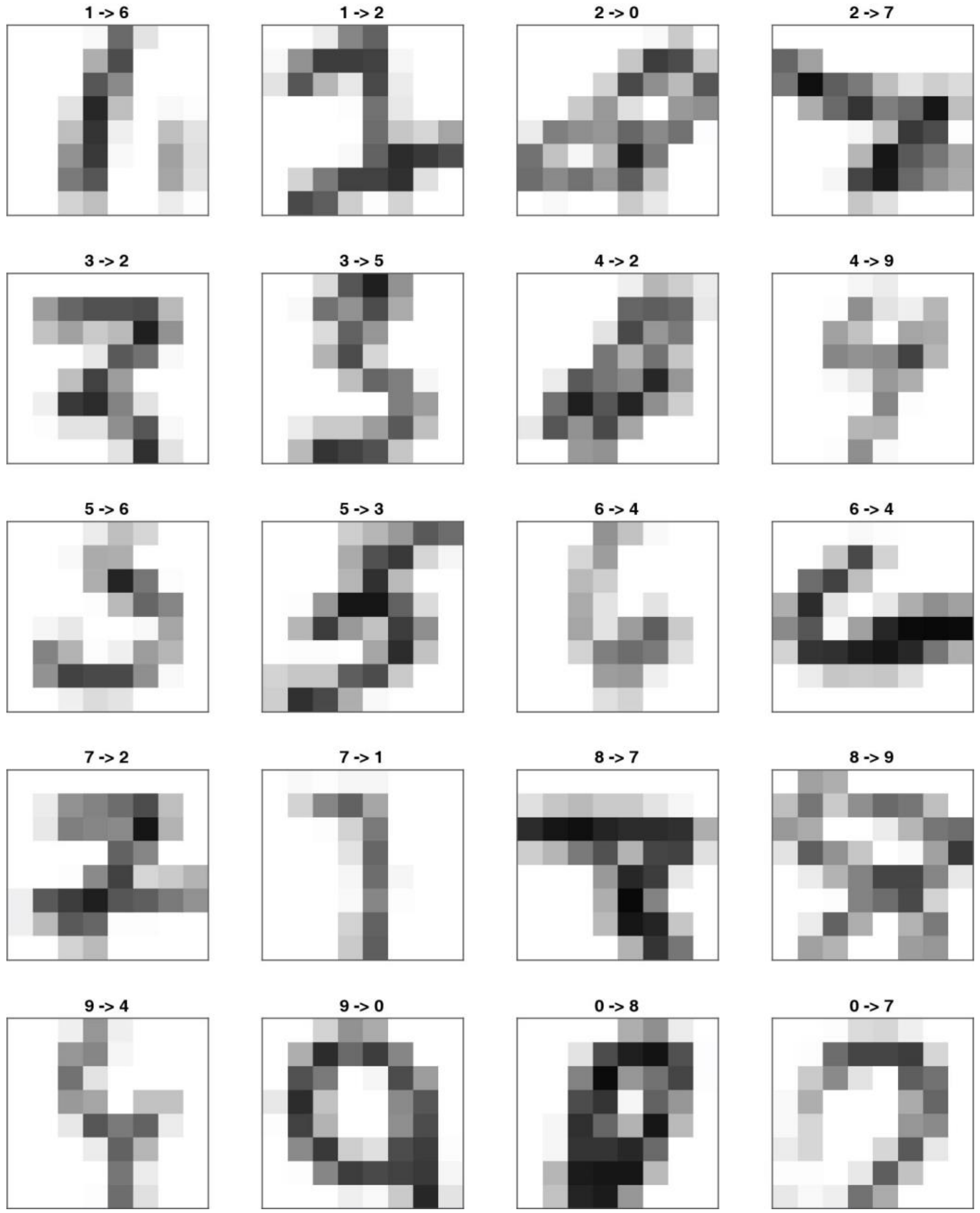

Supplementary Figure 10. Typical MNIST digit images which were misclassified (two typical images for each digit). The title of the images shows the correct label of the image (left to the arrow) and the experimental classification result (right to the arrow).

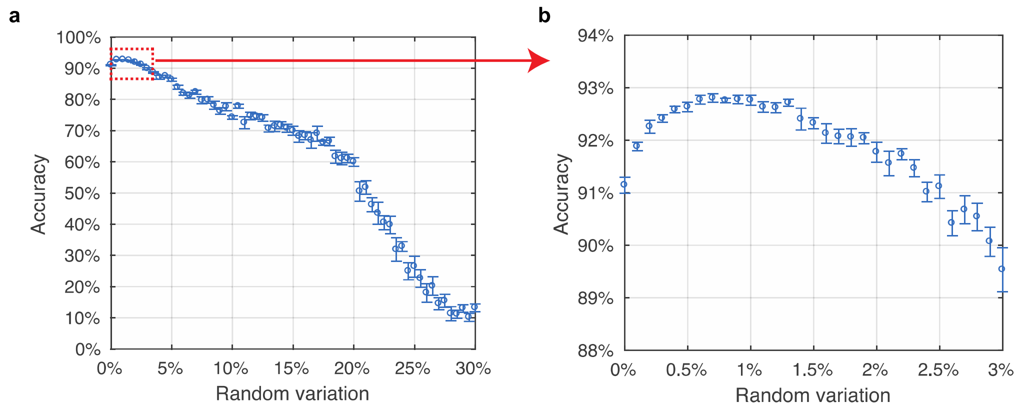

Supplementary Figure 11. Simulated impact on device variation after conductance blind update. a, The impact of random variation after the conductance (i.e. weight) update. Relative random variation is defined as the ratio of conductance update error to the present conductance (σ_G/G). With 30% variance, classification accuracy becomes indistinguishable from random chance. **b,** On the other hand, small amounts of variation (around 1%) improve final classification accuracy relative to extremely low-variance systems. This result shows that a reasonably low-variance weight update scheme, like the one we have implemented using transistors, is very important. On the other hand, small amounts of random variation arising from the intrinsic stochasticity of memristor dynamics do not seem to pose a problem for neural networks.

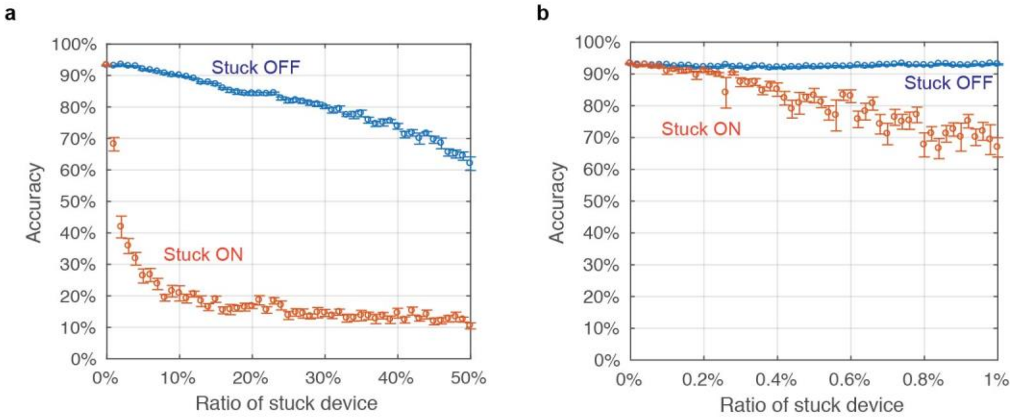

Supplementary Figure 12. Simulated impact on training accuracy from unresponsive devices that are stuck in different conductance state. Blue curves show the impact of devices stuck in a very low-conductance state (10 µS, OFF state), while red shows that of devices stuck at a very high-conductance state (5 mS, ON state). These high- and low-conductance values are typical of device failures observed in the experiment. The tolerance for stuck OFF devices is much greater than that for stuck ON devices. **a,** With 50% stuck OFF devices, the network can still achieve 60% accuracy. **b,** It takes only 1% of devices stuck ON to see comparable performance degradation. This may be because the output current is too high, which could conceivably be mitigated by rescaling the loss function accordingly (k value in the softmax function (Eq. 3)). However, stuck OFF devices appear to be much more common in practice than their high-conductance counterparts.

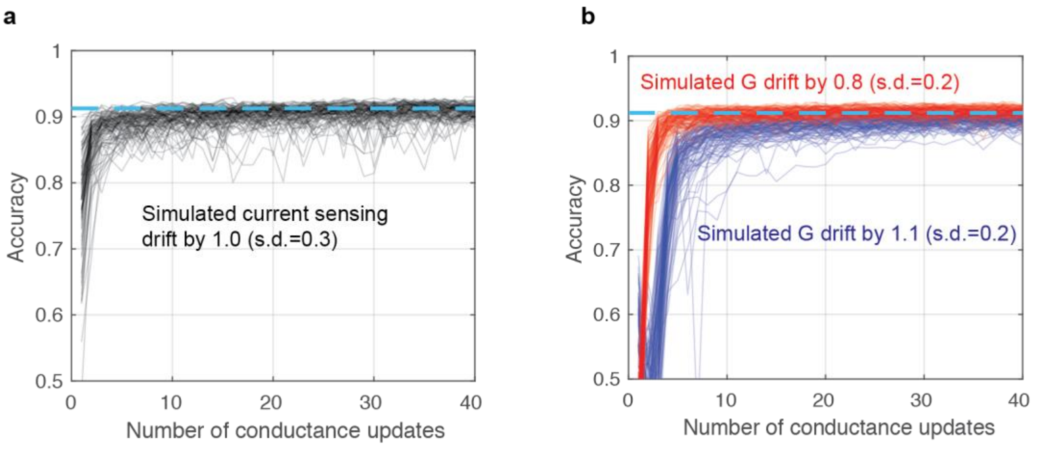

Supplementary Figure 13. Simulated self-adaption to hardware and memristor conductance drift. a, The in-situ training of the memristor network adapts a simulated change in current sensing. We changed a different scaling factor with mean of 1.0 and s.d. of 0.3 on each dimension of the output vector, to simulate a possible hardware drift of the transimpedance amplifier (TIA). The 100-repeated simulation result shows the training algorithm adapts the peripheral asymmetry after training on several samples. The cyan dashed line shows the accuracy before the hardware drift. **b,** The in-situ training of the network adapts the memristor conductance drift. The simulated array was constructed using the same parameters from the experiments, and before the conductance drift the network accuracy is indicated by the cyan dashed line. After the conductance changes by the factor with mean of 0.8 (red)/1.1 (blue) and standard deviation (s.d.) of 0.2 (to simulate a pretty bad case), the in-situ training of the network quickly adapts after training a few samples (plot shows 100 repeated simulation). It is noteworthy that the amount of the simulated conductance drift is unlikely in an experimental array, but we simulated a worse case to make the self-adaption process more noticeable.

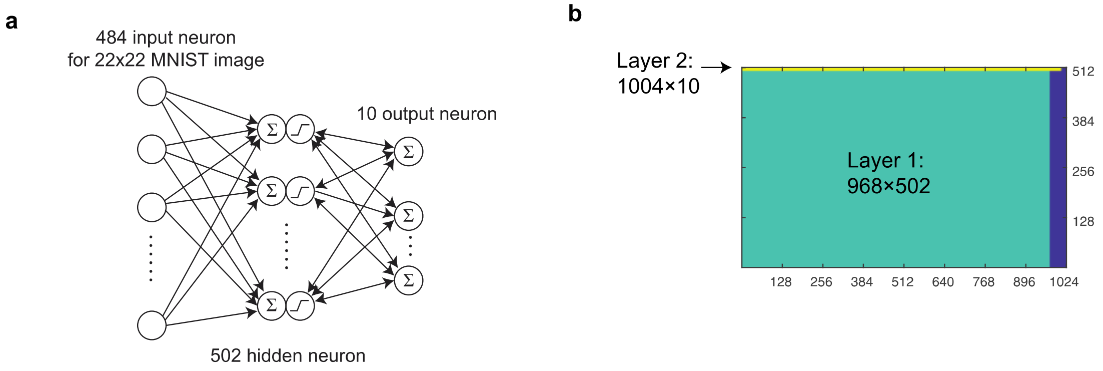

Supplementary Figure 14. A simulated two-layer fully connected network on a large array. a, The network structure that consist of 484 input neurons (for 22×22 images cropped from 28×28 MNIST handwritten digits image), 502 hidden neurons, and 10 output neurons. **b**, The network simulated on a 512 × 1024 memristor crossbar array, with the first layer consist of 968×502 memristors (two memristor differential conductance represents one synaptic weight), and the second layer consist of 1004×10 memristors.

Supplementary Table

Supplementary Table 1. Classification results on the 10,000 separate testing images after training on 80,000 images. Table showing the most-active neuron vs identified input digit, summed over all 10,000 images in the test set. The most common misidentifications involved the digit ‘3’, which was mistaken for an ‘8’ or a ‘5’ on 40 occasions each. The overall accuracy was 91.71%.

	actual 0	actual 1	actual 2	actual 3	actual 4	actual 5	actual 6	actual 7	actual 8	actual 9	precision
predicted 0	953	0	16	6	1	16	19	6	7	4	92.7%
predicted 1	0	1112	2	1	1	3	4	8	4	11	97.0%
predicted 2	5	5	943	25	15	13	17	37	14	2	87.6%
predicted 3	2	2	5	874	1	18	1	5	13	12	93.7%
predicted 4	2	0	10	0	883	5	11	9	6	28	92.6%
predicted 5	5	0	2	40	3	795	14	0	17	16	89.1%
predicted 6	3	5	9	4	10	12	884	0	4	14	93.5%
predicted 7	5	1	15	12	7	2	1	925	3	16	93.7%
predicted 8	5	10	27	40	10	23	7	8	900	8	86.7%
predicted 9	0	0	3	8	51	5	0	30	6	898	89.7%
											Accuracy
recall	97.2%	98.0%	91.4%	86.5%	89.9%	89.1%	92.3%	90.0%	92.4%	89.0%	91.7%

Supplementary Notes

Supplementary Note 1

Main loop of the training algorithm:

```
        % The main training loop:
            for j = 1:n_batches % For each batch

                % Perform inference in the crossbar
                batch = data(:,(j-1)*batch_size+1:j*batch_size);
                [outs, voltages] = obj.test(batch);

                grad = z; % Initialize cumulative gradient to all zeros

                for k=1:batch_size
                 %For each example, compute the gradient estimate by backpropagation in software:
                    I_out = outs(:,k);
                    indx = (j-1)*batch_size+k; % Compute index of this training example

                    % Get voltages for this example
                    V = cellfun(@(x) x(:,k), voltages, 'UniformOutput', false);
                   % Get gradient based on this example
                    temp = obj.calculate_update(V, I_out, labels(indx), w);
                   % Add it to the cumulative batch gradient
                    grad = cellfun(@plus, grad, temp,'UniformOutput',false);
                end

                % Apply the weight update in crossbar:
                obj.update_weights(grad, rate/batch_size, j);
                [w, rawG] = obj.get_weights('mode','fast'); % Read new weights
          end
```

Forward pass:

```
          for i=1:obj.n_layers % For each layer
               if any(abs(voltages{i}(:)) > obj.V_read)
                    % Clipping signals to protect the array operation
                    voltages{i}(voltages{i} > obj.V_read) = obj.V_read;
                end

                [currents{i},raw_out{i}] = obj.array.subs{i}.read_current([voltages{i}; -voltages{i}]);
                voltages{i+1} = obj.activation(currents{i});
          end
            I_out = currents{end};
```

The software implementation of ReLU and its derivative:

```
        c = 200; % The scaling factor that convert a current to a voltage
        activation = @(x) c*max(0, x);   % ReLU
        aderiv     = @(x) c*(x>0);               % derivative of ReLU
```

Backward pass:

```
            p = zeros(1,length(I_out));
            for i=1:length(I_out)
                p(i) = 1./sum(exp(k*(I_out-I_out(i)))); % Softmax
            end

            deltas{end} = -p;
            deltas{end}(label) = (1-p(label));

            for i=numel(deltas):-1:1
                grad{i} = (voltages{i}*deltas{i})'; % Transpose to line up with weights
                if i>1
                    deltas{i-1} = ((deltas{i} * weights{i}) .* obj.aderiv(voltages{i}'));
                end
            end
```
