# Supplementary material for: Efficient and self-adaptive in-situ learning in multilayer memristor neural networks
Source: Nat Commun. 2018 Jun 19;9:2385. doi: 10.1038/s41467-018-04484-2 (PMC6008303; doi:10.1038/s41467-018-04484-2)
Supplement: Supplementary file 3 — Description of Additional Supplementary Files [file 41467_2018_4484_MOESM3_ESM.pdf]

Description of Additional Supplementary Files

File Name: Supplementary Movie 1
Description: The in-situ online training process in the memristor crossbar array. Left panel shows the synaptic weights in the first layer during the training and each weight is represented by the conductance different of two memristors. The weights are readout and arranged in the same way as in the Supplementary Fig. 8. Upper right panel shows the second layer synaptic weights. Lower right panel shows the online accuracy corresponding the current weight shown in the left and upper layer panels.

File Name: Supplementary Movie 2
Description: Another 100 inference samples for hand written digit recognition. The data is arranged in the same way as in the Supplementary Fig. 7. This inference animation is slowed down for human eye inspection.

File Name: Supplementary Movie 3
Description: Real-time inference animation. The memristor based system completed 10,000 handwritten digit recognitions in about 4 mins. Some samples are skipped in this video because the limit of the frame rate (30 frames per second).
